# Asiatic Acid Alleviates Renal Damage by Upregulating STBD1-Mediated Glycophagy in Diabetic Kidney Disease

**DOI:** 10.3390/biomedicines13071544

**Published:** 2025-06-25

**Authors:** Lei Guo, Peili Wu, Qijian Feng, Xiaochun Lin, Yuan Wang, Minghai Wu, Feifei Cai, Jin Zhang, Chuyi Yang, Xuelin Li, Churan Wen, Yingbei Lin, Nannan Liu, Yuxuan Hu, Huiyun Wang, Xinzhao Fan, Meiping Guan

**Affiliations:** 1 Department of Endocrinology and Metabolism, Nanfang Hospital, Southern Medical University, Guangzhou 510515, China; 2 Department of Endocrinology and Metabolism, Zhujiang Hospital, Southern Medical University, Guangzhou 510280, China

**Keywords:** diabetic kidney disease, asiatic acid, glycogen metabolism, STBD1, glycophagy

## Abstract

**Background/Objectives:** The role of glycogen metabolism in diabetic kidney disease (DKD) remains unclear. This study investigated the therapeutic potential of asiatic acid (AA) on glycogen metabolism in DKD and its underlying mechanisms. **Methods:** A DKD mouse model was established using a high-fat diet and streptozotocin, followed by AA treatment for 8 weeks. Network pharmacology and molecular docking identified STBD1 as a potential target of AA, and its overexpression in mice was performed. **Results:** AA reduced blood glucose levels and the urinary albumin-to-creatinine ratio (UACR) and downregulated TGFβ-1, KIM-1, and PDK4. Additionally, AA treatment reversed abnormal glycogen accumulation and restored STBD1 expression. Network pharmacology and molecular docking identified STBD1 as a potential target of AA, and its overexpression in mice demonstrated similar beneficial effects. Gene enrichment analysis revealed that STBD1 is involved in key metabolic pathways related to DKD. **Conclusions:** These findings suggest that AA alleviates renal damage in DKD, possibly through modulation of STBD1, highlighting its therapeutic potential and the critical role of STBD1 in renal glycophagy.

## 1. Introduction

Diabetic kidney disease (DKD) is a prevalent and debilitating condition, emerging as a leading cause of end-stage kidney disease (ESKD) globally and imposing a substantial public health burden [1]. The kidneys are pivotal in glucose homeostasis, and there has been a recent resurgence in interest regarding the role of glucose metabolism in DKD, especially with the introduction and extensive study of sodium-glucose cotransporter 2 inhibitors (SGLT2i) [2]. This renewed interest underscores the imperative for a more profound comprehension of the complex mechanisms that regulate carbohydrate metabolism, which are integral to the pathogenesis and progression of DKD [3].

Asiatic acid (AA), a pentacyclic triterpenoid derived from the Centella asiatica plant, which is revered in traditional medicine, exhibits properties that are anti-inflammatory, antioxidant, and anti-apoptotic [4]. The renoprotective properties of AA, alongside its efficacy against a myriad of ailments, have garnered considerable interest and scrutiny from researchers [5,6,7,8]. Recent research indicates that AA may provide a protective effect on ischemic cardiomyocytes, modulating energy metabolism pathways that encompass both glycophagy and mitochondrial autophagy [9]. These findings prompt the intriguing hypothesis that AA could have analogous metabolic regulatory effects within the context of DKD.

Metabolic reprogramming plays an increasingly recognized role in the pathophysiology of DKD [10]. A particularly intriguing aspect is the abnormal accumulation of glycogen within renal tissue, such as the “Armanni–Ebstein lesions” seen in poorly controlled diabetes [11]; these highlight its potential importance in disease progression [12]. While glycogen serves as an essential energy reserve in normal physiological conditions, reports indicate abnormal deposition of glycogen in the kidneys during DKD, suggesting a potential derangement in glycogen synthesis, degradation, or both [13,14]. Despite these insights, the specific mechanisms behind glycogen dysregulation in DKD and its impact on metabolic reprogramming are not well understood, mandating further investigation.

In recent years, starch-binding domain-containing protein 1 (STBD1), also referred to as Genethonin-1, has been recognized as a novel facilitator of glycophagy, which is the autophagic degradation of glycogen [15]. Glycophagy, a unique alternative to the traditional cytoplasmic glycogen degradation pathway, involves the degradation of glycogen within autophagic vesicles [16,17,18]. This process releases free glucose, providing a quick energy source for cells with high energy demands [19]. STBD1 operates as a cargo receptor that aids in the anchoring of glycogen to lysosomes, a critical step in the glycophagic process [20]. Given its critical role in glycogen turnover and metabolism [21], its potential involvement in the pathophysiology of DKD is both intriguing and underexplored, meriting further investigation to clarify its possible role in the glycogen anomalies associated with this condition.

Collectively, this study endeavors to delineate the altered landscape of glycogen metabolism within DKD and to further investigate the therapeutic potential of AA in mitigating DKD through the modulation of STBD1-mediated metabolic pathways.

## 2. Materials and Methods

### 2.1. Animal Models

Male C57BL/6J mice, aged 6–8 weeks, purchased from the Animal Research Center of Southern Medical University, were maintained at 22 °C with a 12 h light/dark cycle with free access to water and standard chow or special diets, unless otherwise noted. For the HFD/STZ-induced DKD model, 8-week-old mice were fed a high-fat diet (D12492, 60% fat, Research Diets, New Brunswick, NJ, USA) for 5 weeks and then received intraperitoneal Streptozotocin (STZ) injections (50 mg/kg per day) for 5 days. For the STZ-induced DKD model, 8-week-old mice received a single injection of STZ (150 mg/kg). Male C57BLKS/J diabetic mice (db/db; 6 weeks) and male C57BLKS/J normal mice (db/m; 6 weeks) were purchased from the Nanjing Model Animal Centre. The AA treatment group was given AA (10 mg/kg, 98%, Aladdin Biochemical Technology Co., Ltd., Shanghai, China.) by gavage, and the control group and model group were given the same volume of saline by gavage for 8 weeks. For in vivo STBD1 overexpression, adeno-associated virus encoding STBD1 (AAV9-STBD1) purchased from Genechem (Shanghai, China) was delivered by tail vein injection. For the fasting, mice were maintained in a standard housing environment for 24 h, with unrestricted access to water. All animal use protocols were reviewed and approved by the Animal Experimental Committee of Nanfang Hospital.

### 2.2. Glucose Sensitivity and Insulin Tolerance Testing

An intraperitoneal glucose tolerance test (IPGTT) and intraperitoneal insulin tolerance test (IPITT) were conducted as previously described [22].

### 2.3. Blood Glucose and Urinary Albumin-to-Creatinine Ratio (UACR)

The blood glucose levels were determined using an automated blood glucose meter (Sannuo, Changsha, China). Urinary albumin and creatinine were assayed using a mouse albumin ELISA kit (Meimian Industrial Co., Ltd., Yancheng, China) and urinary creatinine assay kit (Nanjing Built Biological Technology Co., Ltd., Nanjing, China) according to the manufacturer’s instructions.

### 2.4. Reverse Transcription-Quantitative PCR (RT-qPCR) and Western Blot Analysis

Total RNA was extracted using Trizol reagent (Thermo Fisher, Waltham, MA, USA). cDNA was synthesized using a PrimeScript RT Reagent Kit (Ecory Bioengineering Co., Ltd., Nanjing, China). RT-qPCR was performed using a SYBR Real-time PCR Master Mix kit (Yeasen Biotechnology Co., Ltd., Shanghai, China) on ABI QuantStudio™ 5 system (Thermo Fisher Scientific, Waltham, MA, USA). The expression levels were analyzed by the 2^−ΔΔCt^ method. Protein expressions were detected by western blot (WB), as previously reported [23]. Additional details can be found in Tables S1 and S2.

### 2.5. Glycogen Detection

Glycogen staining was performed with a Periodic Acid-Schiff (PAS) kit (Sigma-Aldrich, St. Louis, MO, USA), and the amount of glycogen in kidney tissues was quantified using a glycogen content assay kit (Nanjing built Biological Technology Co., Ltd., Nanjing, China) according to the manufacturer’s instructions.

### 2.6. Electron Microscopy

The microstructure was observed by transmission electron microscopy. In brief, kidney tissue was fixed with 2.5% glutaraldehyde, then infiltrated with 1% osmium tetroxide in cacodylate buffer, dehydrated, and embedded in epoxy resin. The samples were sectioned and stained with 2% uranyl acetate and lead citrate, and images were collected using a Tecnai G 2 20 TWIN transmission electron microscope (FEI Company, Hillsboro, OR, USA).

### 2.7. Immunohistochemistry

Immunohistochemical analysis was conducted according to established methodologies. In brief, renal tissues were initially deparaffinized using xylene and subsequently rehydrated through a graded series of ethanol washes. Endogenous peroxidase activity was quenched with a 3% hydrogen peroxide solution. Antigen retrieval was facilitated by immersing the samples in a boiling citric acid solution with a pH of 6.0. Non-specific binding was minimized by pre-incubating with blocking serum matching the host species of the secondary antibody. Thereafter, the tissues were exposed to anti-STBD1 primary antibodies (diluted 1:200) and incubated at 4 °C overnight. Post incubation, the slides were treated with biotinylated secondary antibodies (diluted 1:100), followed by incubation with horseradish peroxidase (HRP)-conjugated streptavidin. Chromogenic detection was achieved using a diaminobenzidine (DAB) substrate kit (DAKO).

### 2.8. Hematoxylin-Eosin (HE) Staining and Masson Staining

The formalin-fixed, paraffin-embedded samples were sliced into 5 μm thick sections. HE and Masson staining of mice sections were performed using an HE staining kit and Masson Trichrome Staining Kit according to the manufacturer’s instructions (Hundred Thousand Degree Biotechnology Co., Ltd., Wuhan, China). Images per slide were captured using a microscope (Olympus BX63, Olympus, Tokyo, Japan).

### 2.9. Molecular Docking Analysis

Molecular docking was performed with Autodock software v1.2.5 [24]. The 3D structure of STBD1 was sourced from the AlphaFold Protein Structure Database (STBD1_AF_O95210_F1_model_v4_model1), while the 3D structure of AA was retrieved from the NCBI PubChem Compound Summary for CID 119034. Before docking, the protein and ligand structures were prepared and saved in PDBQT format. The docking grid was centered at X = −2.149, Y = 4.072, Z = −2.793. Molecular docking was visualized using PyMOL 2.5.

### 2.10. Network Pharmacology

Genes associated with AA and DKD were collected from multiple databases, including CTD, GeneCards, SwissTargetPrediction, and OMIM. Common genes between AA and DKD were identified using a Venn diagram, with differential gene expression visualized via a heatmap. KEGG pathway enrichment analysis was performed to identify significant biological pathways related to differentially expressed genes (DEGs), with the results displayed in a bubble chart showing gene count and significance. A protein–protein interaction (PPI) network was constructed and analyzed using Cytoscape v3.7.2, with top biological processes and molecular functions derived from the GO database and visualized in a bar chart. Statistical significance was assessed using the hypergeometric test, followed by Benjamini–Hochberg adjustment for multiple testing, with *p*-values < 0.05 considered statistically significant.

### 2.11. Gene Set Enrichment Analysis

The GSE228727 dataset [25] was employed to explore the role of STBD1 in DKD. We constructed a PPI network to identify pivotal interactions involving STBD1 and performed gene set variation analysis (GSVA) alongside gene set enrichment analysis (GSEA) to uncover metabolic pathways enriched and associated with STBD1 expression. For each analysis, statistical significance was assessed using suitable methods, with *p*-values less than 0.05 indicating significant results.

### 2.12. Statistical Analyses

Statistical analysis was performed using SPSS version 25.0. The experimental data were represented by mean ± standard error of the mean, double-tail *t*-test was used for data analysis between two groups, one-way ANOVA was used for comparison between three groups or more, and Tukey test was used for comparison between groups. *p* < 0.05 was considered statistically significant.

## 3. Results

### 3.1. AA Improves Blood Glucose and UACR in DKD

**Figure 1:**
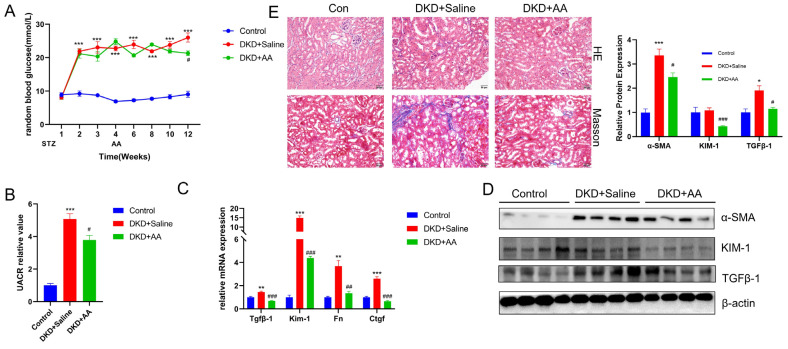
Protective effects of AA treatment on RBG, UACR, and renal phenotype in DKD. (**A**) Random blood glucose (RBG) in each group of mice. (**B**) UACR of mice in each group. (**C**,**D**) Alterations in renal indices after AA treatment. (**E**) Representative kidney histology showing HE and Masson stains (scale bar = 50 μm). *, *p* < 0.05; **, *p* < 0.01; ***, *p* < 0.001 vs. control. #, *p* < 0.05; ##, *p* < 0.01; ###, *p* < 0.001 vs. DKD + saline (*n* = 4).

The results showed that random blood glucose (RBG) and UACR were significantly elevated after DKD modeling compared to control mice. After 8 weeks of AA treatment, the increases in RBG and UACR were significantly improved (Figure 1A,B). Furthermore, RT-qPCR analysis revealed KIM-1 expression (a kidney injury marker), along with fibrosis-related genes (Fn and Tgfβ1), was significantly increased in DKD + saline and reduced by AA treatment (Figure 1C). Western blot confirmed corresponding changes in the corresponding proteins (Figure 1D). In Figure 1E, the pathological staining also demonstrated that AA treatment improved the tissue pathological damage and collagen deposition in the kidney of DKD mice. Taken together, these results indicate a renoprotective effect of AA in DKD.

### 3.2. AA Modulates Kidney Glycogen Metabolism in DKD

**Figure 2:**
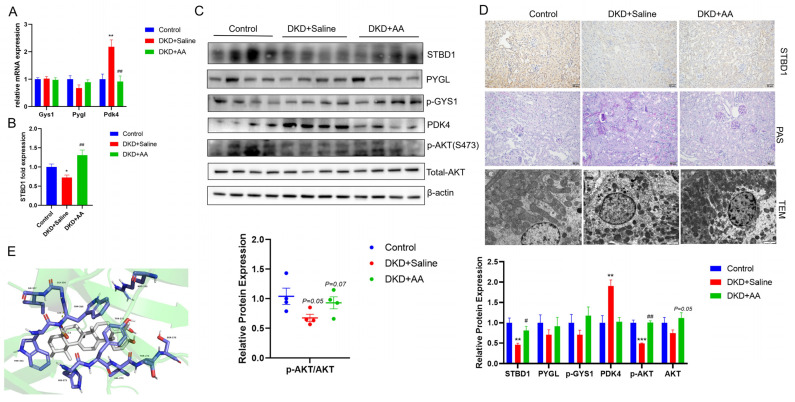
Effect of AA treatment on renal glycogen metabolism. (**A**–**C**) Altered glycogen metabolism after AA treatment. (**D**) Immunohistochemistry of STBD1 and glycogen deposition by PAS staining (scale bar = 50 μm) and TEM microscopy (scale bar = 2 μm). (**E**) Molecular docking of AA with STBD1. *, *p* < 0.05; **, *p* < 0.01; ***, *p* < 0.001 vs. control. #, *p* < 0.05; ##, *p* < 0.01 vs. DKD + saline (*n* = 4).

Compared to the DKD + saline group, no significant differences were observed in the expression of key proteins involved in glycogen synthesis, GYS1, or the classical cytoplasmic degradation pathway, PYGL, after AA treatment (Figure 2A,C). Notably, there was a pronounced upregulation of STBD1, a protein integral to an alternative glycogen degradation pathway (Figure 2B–D). Concurrently, we also identified alterations in the expression of PDK4, a critical nexus in glycolipid metabolism (Figure 2A,C). Western blot analysis further confirmed the activation of the AKT signaling pathway post AA administration (Figure 2C). In Figure 2D, AA treatments also improved the abnormal glycogen deposition in the kidney of DKD compared to DKD + saline. Synthesizing these observations, our results imply that the renoprotective action of AA might be mediated, at least in part, through the upregulation of STBD1 consequent to the activation of the AKT pathway. In addition, using STBD1 as the receptor and AA as the ligand, the molecular docking results showed an optimal binding energy of −7.129 kcal/mol, indicating good binding activity between the two. The interaction site is shown in Figure 2E.

### 3.3. Network Pharmacology of AA and DKD

**Figure 3:**
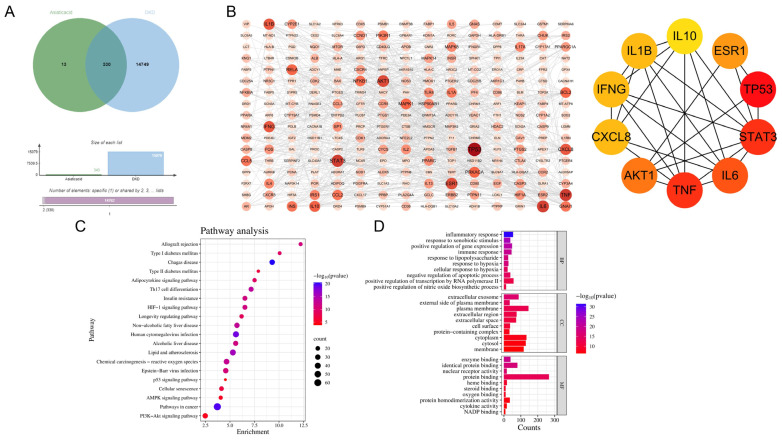
Network pharmacology of AA and DKD. (**A**) Venn diagram showing the intersection of genes related to AA and DKD. (**B**) PPI and core target. (**C**,**D**) Analysis of GO and KEGG enrichment.

To further validate the potential core targets of AA in DKD, we conducted a network pharmacological analysis. The Venn diagram (Figure 3A) revealed that 330 genes were common between the sets associated with AA and DKD. The PPI network (Figure 3B) constructed from these overlapping genes highlighted key nodes such as IL6, AKT1, and TNF, indicating their central roles in the network. Pathway enrichment analysis (Figure 3C) showed that significantly enriched pathways included inflammatory response, immune response, and cellular processes. Functional annotation clustering (Figure 3D) further illustrated that the significant functional annotations were primarily related to inflammation, immune response, and cellular signaling pathways. These findings exhibit strong alignment with our previous conclusions, leading us to hypothesize that AA likely ameliorates DKD by modulating metabolic pathways, particularly the STBD1-mediated glycogen metabolism pathway, which, in turn, regulates downstream inflammatory and fibrotic cascades.

### 3.4. Glycogen Deposition in DKD

**Figure 4:**
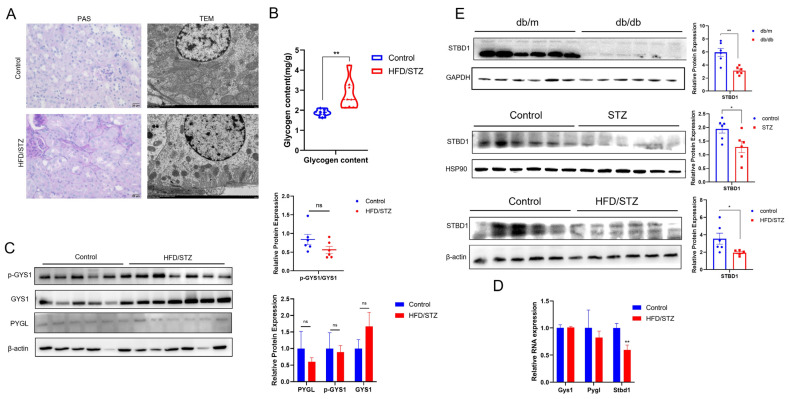
Glycogen deposition and metabolism in the kidney of DKD. (**A**) Glycogen deposition observed by PAS and TEM (PAS, scale bar = 20 μm; TEM, scale bar = 2 μm). (The HFD/STZ group refers to the DKD + saline group mentioned earlier in the text, so it is the same field as in Figure 2D: DKD kidney, high magnification.) (**B**) Glycogen content determination. (**C**,**D**) Glycogen metabolism in DKD. (**E**) Detection of STBD1 in the kidney of three distinct diabetic mouse models. *, *p* < 0.05; **, *p* < 0.01 vs. control. (**E**) **, *p* < 0.01 vs. db/m (*n* = 5–6).

To further validate, we explored glycogen metabolism in DKD. In the animal model of DKD, histological examination via PAS staining demonstrated markedly enhanced glycogen deposition within the renal cortices of the DKD group when juxtaposed with control groups. This was further supported by electron microscopy, which revealed abnormal glycogen accumulations within the cytoplasm of renal cells (Figure 4A). Furthermore, the quantitative analysis of glycogen content in renal tissue confirms a trend toward increased glycogen accumulation in the DKD state (Figure 4B). Subsequent RT-qPCR and WB analyses showed a downregulation of STBD1, an indicator involved in glycophagy (Figure 4C,D). Interestingly, a consistent and significant downregulation of STBD1 in the kidney was observed across all three distinct diabetic mouse models (Figure 4E). In contrast, no significant changes were observed in the levels of key genes and proteins associated with glycogen synthesis and cytoplasmic degradation pathways (Figure 4C,D). Taken together, these results suggest that the abnormal glycogen accumulation observed in DKD may be due to the inhibition of the glycophagy pathway, which regulates glycogen degradation.

### 3.5. Modulatory Effects of STBD1 Overexpression in DKD

**Figure 5:**
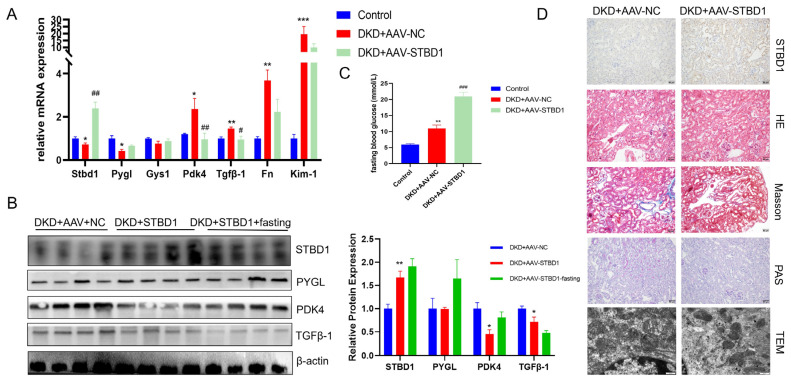
Effect of STBD1 overexpression in DKD. (**A**,**B**) Alterations in associated genes and proteins after overexpression of STBD1. (**C**) Fasting blood glucose in each group of mice after 24 h of fasting. (**D**) Histopathological and ultrastructural changes after STBD1 overexpression (stains, scale bar = 50 μm; TEM, scale bar = 500 nm). *, *p* < 0.05; **, *p* < 0.01; ***, *p* < 0.001 vs. control. #, *p* < 0.05; ##, *p* < 0.01; ###, *p* < 0.001 vs. DKD+AAV-NC. (**B**) *, *p* < 0.05; **, *p* < 0.01 vs. DKD+AAV-NC (*n* = 4).

**Figure 6:**
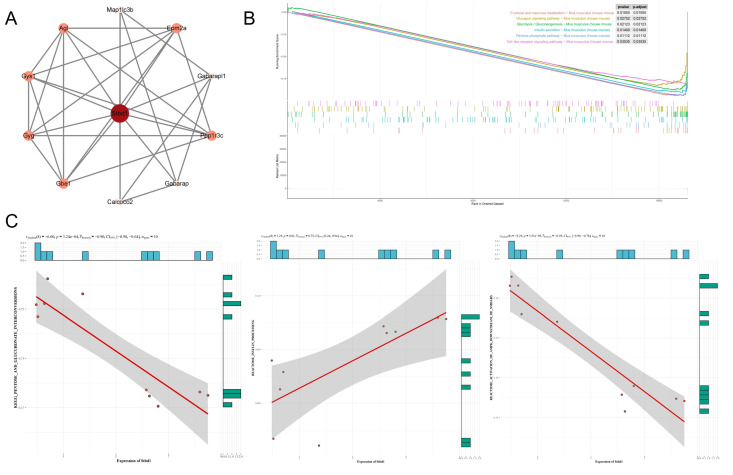
STBD1 PPI network and enrichment analysis. (**A**) STBD1 PPI network. (**B**) GSEA results. (**C**) From left to right: the correlation between STBD1 and the pentose–glucuronate interconversion pathway, AMPK activation downstream of the NMDAR signaling pathway, and insulin processing pathway. GSEA, gene set enrichment analysis; GSVA, gene set variation analysis.

To further validate how glycogen metabolism affects DKD, we delved into the key protein STBD1 identified in our prior findings. We examined the expression of STBD1 in major organs under DKD conditions (Figure S1). The consistent downregulation of STBD1 within the renal tissues of three distinct diabetic mouse models prompted us to conduct in vivo studies with STBD1 overexpression. As depicted in Figure 5, tail vein injection of AAV9-STBD1 resulted in elevated renal expression of STBD1. In mice with elevated STBD1 levels, there was a downregulation in the expression of TGFβ-1 and PDK4 when compared to DKD controls (Figure 5A,B). Figure 5D also confirmed the histopathological and ultrastructural changes in the kidneys of DKD mice following STBD1 overexpression. Of particular interest, STBD1-overexpressing mice displayed markedly higher fasting blood glucose levels (Figure 5C), whereas the levels of random blood glucose were unaltered in comparison to DKD controls (Figure S2), hinting at a possible modulation of insulin signaling by STBD1 overexpression. We additionally acquired the GSE228727 dataset for complementary analysis. Figure 6A presents a PPI network centered on STBD1, highlighting interactions with proteins like Map1lc3b, Gys1, Gabarapl1, and Calcoo2. Figure 6B shows pathway enrichment results from the GSEA, revealing significant enrichment of pathways related to glucose metabolism, insulin signaling, and inflammation, including “fructose and mannose metabolism,” “glycolysis/gluconeogenesis,” and “insulin secretion” (*p* < 0.05). These findings suggest a strong link between STBD1 and metabolic pathways disrupted in DKD. Figure 6C presents a correlation analysis of STBD1 expression with various metabolic pathways, showing significant correlations, further supporting its role in metabolic dysregulation in DKD. More details are available in Figure S3.

## 4. Discussion

The pathophysiology of DKD encompasses a multitude of mechanisms [26], with a notable upsurge in research attention on renal metabolic reprogramming and aberrant metabolic states in recent years [27]. This study directs particular attention to the heretofore underappreciated issue of abnormal glycogen deposition within the renal tissues of DKD, a condition potentially attributable to the suppression of glycogen catabolic pathways. Concurrently, our research implicates AA, an ingredient derived from traditional Chinese medicine, as a prospective renoprotective agent, with its mechanism of action possibly entailing the upregulation of STBD1 to rectify the dysregulated renal metabolism associated with DKD. These insights not only illuminate the intricate dynamics of glucose metabolism associated with DKD but also lay the groundwork for the development of targeted therapeutic strategies aimed at alleviating DKD.

AA, a pentacyclic triterpene derived from the traditional herbal remedy Centella asiatica, has anti-inflammatory, antioxidant, and anti-apoptotic properties. Antioxidants seem to have therapeutic effects in patients with DKD, especially in reducing proteinuria and HbA1c [28]. Previous studies have also demonstrated its nephroprotective effects through inflammation and oxidative stress-mitigating pathways [6,29]. Qiu F et al. reported that AA protects ischemic cardiomyocytes by orchestrating glycophagy- and mitophagy-driven energy metabolism via the PI3K/Akt and AMPK signaling axes [10]. Similarly, our study results indicate that the common predicted targets of AA and DKD are primarily concentrated in pathways related to inflammation, immunity, and metabolism. Our in vivo experiments also corroborate these findings. Additionally, we discovered that, in addition to upregulating STBD1 via the AKT pathway, AA may also act directly on the STBD1 molecular site, representing another previously unrecognized mechanism of renoprotection. Despite demonstrating numerous therapeutic benefits, the clinical application of AA is currently constrained by its unfavorable physicochemical properties [30]. However, promising research is focused on developing targeted drug delivery systems, including nanoparticle-based systems [31]. Future studies that delve deeper into the mechanisms of action and innovative delivery methods for AA are expected to broaden its clinical applications significantly.

The kidneys play a vital role in maintaining glucose homeostasis, but their ability to regulate glycogen synthesis, storage, and degradation is impaired in DKD, exacerbating renal dysfunction [12]. It has long been known that glycogen is virtually absent in healthy kidney tissue; however, abnormal accumulation of this macromolecule is consistently observed in diabetic kidneys [11,14,15,32,33]. Our findings, supported by both glycogen content measurements and PAS staining, as well as electron microscopy, are consistent with this prevailing study and collectively provide evidence for the presence of abnormal glycogen deposition in DKD. However, the underlying cause of this glycogen dysregulation remains controversial and urgently needs to be explored in depth.

Glycogen granules can be utilized by two pathways: (1) cytosolic degradation by the coordinated action of PYGL and glycogen-debranching enzyme (GDE), and (2) lysosomal degradation by the action of STBD1 and α-glucosidase [17]. Although the former is widely recognized, the latter is equally important for the maintenance of glucose homeostasis through its modulation by signaling cascades such as cAMP-PKA/PI3K-Akt/PKB-Mtor [20]. Research has implicated alterations in glycophagy in diverse pathologies, exemplified by the upregulation of STBD1 in diabetic heart mouse models to facilitate the clearance of excess glycogen [34], and its significant and selective association with glycogen accumulation in female myocardium [35], potentially serving as a critical component of cardiac metabolic stress responses [36]. STBD1 also plays a key role in the translocation of hepatic glycogen to lysosomes and the metabolic transition from glycogen to lipids in skeletal muscle [37,38]. Our findings suggest that STBD1 plays a critical role in the pathophysiology of DKD by modulating renal metabolic processes. Specifically, the downregulation of STBD1 may exacerbate abnormal glycogen deposition in DKD, while its upregulation could potentially alleviate DKD-induced renal injury. Moreover, the close association between STBD1 expression and metabolic pathway disorders, observed in the GEO dataset analysis, highlights its involvement in the regulation of glucose metabolism, insulin signaling, and inflammation. This interplay between STBD1 and insulin signaling aligns with similar findings in hepatic studies [39]. However, the crosstalk between STBD1 and renal metabolism, as well as its interactions with metabolic processes in other organs such as the liver, skeletal muscle, and myocardium, still requires further experimental investigation.

Nonetheless, several limitations of this study must be addressed. First, the DKD mouse model employed may not entirely reflect human glycogen metabolism, underscoring the necessity for further validation using clinical specimens to verify the phenotypic results and delve deeper into the underlying mechanisms. Moreover, future research should delve into more complex mechanistic aspects, such as conducting relevant functional experiments to determine the regulatory and binding relationships. It is crucial to evaluate the therapeutic value of different doses of AA and STBD1 across various stages of DKD, as well as to include positive control drug groups. These efforts will enhance our comprehensive understanding of their clinical applications.

## 5. Conclusions

In summary, this study has shed light on the perturbations in glycogen metabolism associated with DKD and furnished novel and initial evidence pointing to a potential renal protective mechanism of AA. This mechanism appears to involve the modulation of STBD1, thereby reshaping the renal metabolic landscape.

## Data Availability

The data used to support the findings of this study are available from the corresponding author upon request.

## Supplementary Materials

The following supporting information can be downloaded at https://www.mdpi.com/article/10.3390/biomedicines13071544/s1, Figure S1: Expression of STBD1 in the heart and liver in DKD mice; Figure S2: STBD1-AAV effect on random blood glucose; Figure S3: STBD1 expression and its associated pathway activity analyzed using GSVA in GEO datasets; Table S1: The primers used in real-time qPCR; and Table S2: Primary antibodies used in western blots.

## Abbreviations

The following abbreviations are used in this manuscript.

AA	Asiatic acid
AKT	Akt (protein kinase B)
DKD	Diabetic kidney disease
ESRD	End-stage kidney disease
FN	Fibronectin
GYS1	Glycogen synthase 1
HFD	High-fat diet
IPGTT	Intraperitoneal glucose tolerance test
IPITT	Intraperitoneal insulin tolerance test
KIM-1	Kidney injury molecule-1
p-GYS1	Phosphorylated glycogen synthase 1
PDK4	Pyruvate dehydrogenase kinase, isozyme 4
PYGL	Glycogen phosphorylase liver
SGLT2i	Sodium-glucose cotransporter 2 inhibitors
STBD1	Starch-binding domain-containing protein 1
STZ	Streptozotocin
TGFβ-1	Transforming growth factor beta-1
UACR	Urinary albumin-to-creatinine ratio
