# Supplementary material for: Asiatic Acid Alleviates Renal Damage by Upregulating STBD1-Mediated Glycophagy in Diabetic Kidney Disease
Source: Biomedicines. 2025 Jun 25;13(7):1544. doi: 10.3390/biomedicines13071544 (PMC12293080; doi:10.3390/biomedicines13071544)
Supplement: Supplementary file 1 [file biomedicines-13-01544-s001.zip › biomedicines-3599348-supplementary.pdf]

Table S1 The primers used in Real-time qPCR

Gene		Primer
m-Stbd1	Forward	TCGAGAAAGCAACGGACATTT
	Reverse	CCACACTGCCAGCTACTTTG
m-Actin	Forward	GTCCACCCCGGGGAAGGTGA
	Reverse	AGGCCTCAGACCTGGGCCATT
m-Gys1	Forward	GAACGCAGTGCTTTTCGAGG
	Reverse	CCAGATAGTAGTTGTCACCCCAT
m-Pygl	Forward	GAGAAGCGACGGCAGATCAG
	Reverse	CTTGACCAGAGTGAAGTGCAG
m-Fn	Forward	ATGTGGACCCCTCCTGATAGT
	Reverse	GCCCAGTGATTTCAGCAAAGG
m-Tgfβ-1	Forward	CCACCTGCAAGACCATCGAC
	Reverse	CTGGCGAGCCTTAGTTTGGAC
m-Pdk4	Forward	AGGGAGGTCGAGCTGTTCTC
	Reverse	GGAGTGTTCACTAAGCGGTCA
m-Kim-1	Forward	ACATATCGTGGAATCACAACGAC
	Reverse	ACAAGCAGAAGATGGGCATTG

Table S2 Primary antibodies used in western blots.

Name	Manufacturer
STBD1	Proteintech (USA)
β-actin	Proteintech (USA)
GYS1	PTM (USA)
p-GYS1	Proteintech (USA)
PYGl	Proteintech (USA)
KIM-1	Affinity (USA)
PDK4	Affinity (USA)
α-SMA	ABclonal (USA)
TGFβ-1	ABclonal (USA)
IL6	ABclonal (USA)
p-AKT(S473)	ABclonal (USA)
AKT	ABclonal (USA)
IgG-HRP	Fude Biological Technology Co., Ltd., (China)

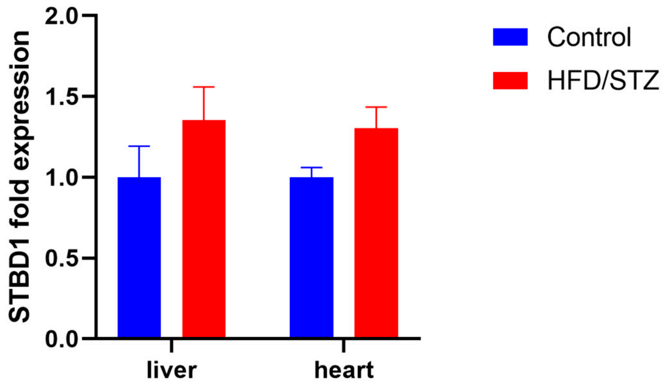

Figure S1 Expression of STBD1 in the Heart and Liver in DKD mice

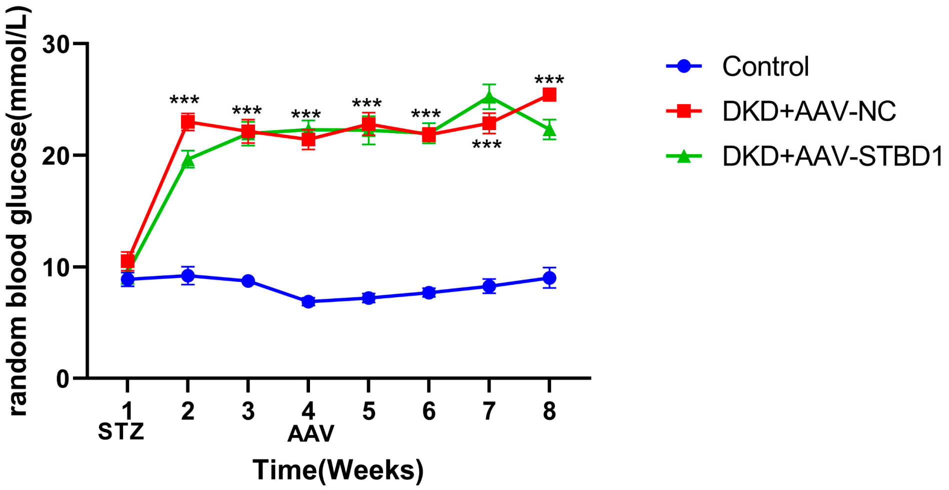

Figure S2 STBD1-AAV effect on random blood glucose

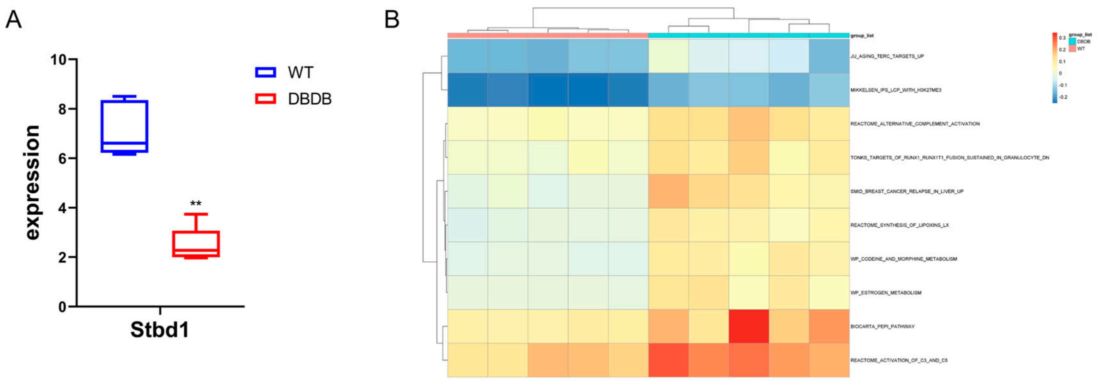

Figure S3 STBD1 expression and its associated pathway activity analyzed using GSVA in GEO datasets
